# Responses of the maize rhizosphere soil environment to drought-flood abrupt alternation stress

**DOI:** 10.3389/fmicb.2023.1295376

**Published:** 2023-12-14

**Authors:** Yun Gao, Yulong Zhao, Ping Li, Xuebin Qi

**Affiliations:** Farmland Irrigation Research Institute of CAAS, Xinxiang, China

**Keywords:** drought-flood abrupt alternation, maize, stress response mechanism, rhizosphere bacteria, soil metabolites, soil enzyme activity

## Abstract

Changes in the soil environment in the root zone will affect the growth, development and resistance of plants. The mechanism underlying the effect of drought and flood stress on rhizosphere bacterial diversity, soil metabolites and soil enzyme activity is not clear and needs further study. To analyze the dynamic changes in bacteria, metabolites and enzyme activities in the rhizosphere soil of maize under different drought-flood abrupt alternation (DFAA) stresses, the barrel test method was used to set up the ‘sporadic light rain’ to flooding (referring to trace rainfall to heavy rain) (DFAA1) group, ‘continuous drought’ to flooding (DFAA2) group and normal irrigation (CK) group from the jointing to the tassel flowering stage of maize. The results showed that Actinobacteria was the most dominant phylum in the two DFAA groups during the drought period and the rewatering period, and Proteobacteria was the most dominant phylum during the flooding period and the harvest period. The alpha diversity index of rhizosphere bacteria in the DFAA2 group during the flooding period was significantly lower than that in other stages, and the relative abundance of Chloroflexi was higher. The correlation analysis between the differential genera and soil metabolites of the two DFAA groups showed that the relative abundance of *Paenibacillus* in the DFAA1 group was higher during the drought period, and it was significantly positively correlated with the bioactive lipid metabolites. The differential *SJA-15* bacterium was enriched in the DFAA2 group during the flooding period and were strongly correlated with biogenic amine metabolites. The relative abundances of *Arthrobacter*, *Alphaproteobacteria* and *Brevibacillus* in the DFAA2 group were higher compared with DFAA1 group from rewatering to harvest and were significantly positively correlated with hydrocarbon compounds and steroid hormone metabolites. The acid phosphatase activity of the DFAA1 group was significantly higher than that of the DFAA2 group during the flooding period. The study suggests that there is a yield compensation phenomenon in the conversion of ‘continuous drought’ to flooding compared with ‘sporadic light rain’, which is related to the improvement in the flooding tolerance of maize by the dominant bacteria Chloroflexi, bacterium SJA-15 and biogenic amine metabolites. These rhizosphere bacteria and soil metabolites may have the potential function of helping plants adapt to the DFAA environment. The study revealed the response of the maize rhizosphere soil environment to DFAA stress and provided new ideas for exploring the potential mechanism of crop yield compensation under DFAA.

## Introduction

Abiotic stresses such as drought, floods, salinity, and high and low temperatures are key limiting variables in modern agriculture that can cause direct or indirect water stress on crops and ultimately affect yield. According to statistics (Statistics Bureau of the People’s Republic of China, 2022), the planting area of maize in China is maintained at approximately 400 billion m^2^, and the total output is maintained at approximately 275 billion kg. As the largest crop in China, there are many climate disasters in maize production, such as drought and flooding in the main producing areas. Drought-flood abrupt alternation (DFAA) mostly occurs from mid-late July to mid-late August, which coincides with the jointing to the flowering stage of maize. The growth and yield of maize are easily affected by DFAA stress. Soil is an environmental carrier for crop growth. Changes in soil microbial diversity, metabolites, and soil enzyme activities indirectly affect aboveground plant growth and yield formation through roots. The rhizosphere is considered the second genome of plants. Rhizosphere soil is a key area for plant growth, nutrient transformation and rhizosphere microbial community development. The decrease in the soil external aeration state under DFAA hinders the growth and reproduction of microorganisms, inhibits the circulation of soil nutrients, and leads to a decrease in the microbial diversity index in soil aggregates. At the same time, flooding stress reduces the ability of soil microorganisms to use carbon sources, mainly amino acids and carbohydrates, resulting in changes in the quantity and quality of belowground carbon input, which further affects the microbiome (Canarini and Feike, 2015; Zhang et al., 2017). Microbial community members enhance the stress resistance of plants by providing buffer zones for plants, producing various hormones that promote plant growth and improving nutrient utilization. Studying the effects of rhizosphere microbial composition, abundance and function on soil metabolism is helpful for exploring the mechanism of the drought and flooding tolerance of crops and improving the adaptability of crops to DFAA stress (Ngumbi and Kloepper, 2016; Castrillo et al., 2017; Nguyen et al., 2018; Rahimi et al., 2020).

The response of rhizosphere microorganisms to single drought and flood stress is different. Studies have found that arbuscular mycorrhizal fungi (AMF) reduce the damage caused by oxidative stress to plants by enhancing the activity of antioxidant enzymes under drought stress and increase the drought tolerance of host plants by increasing plant water use efficiency and biomass (Varoquaux et al., 2019). Plant roots recruit soil microorganisms by accumulating a large amount of glycerol 3-phosphate (G3P) and the stress response factor piperidine acid so that gram-positive bacteria can preferentially colonize to achieve drought resistance (Xu and Coleman-Derr, 2019). Adding Streptomyces will increase the biomass of plant roots, but the exogenous application of iron will reduce the adaptive advantages of these bacteria under drought conditions (Xu et al., 2021). Studies on flooding stress have found that beneficial bacteria that produce 1-aminocyclopropane-1-carboxylic acid deaminase can reduce the stress-induced ethylene content, thereby protecting crops from the harmful effects of flooding, drought and high salt stress. Yonghaparkia can colonize plants to promote the differentiation of rhizobia and can also promote the absorption of iron ions in soil and increase plant resistance (Coutinho et al., 2016; Xu et al., 2018). Under anaerobic conditions, Unclassified-WD2101 performs anaerobic ammonia oxidation, participates in the carbon cycle, promotes the absorption of carbon and nitrogen elements and trace ion elements by crops, and increases the resistance to flooding. The anaerobic heterotrophic growth-promoting bacterium Clostridium colonizes the root system, producing propionic acid and decomposing cellulose, which may help plants obtain more nutrients to adapt to stress; the highly connected microbial community can also stimulate the plant immune system and accelerate the activation of defenses against the outside world, thereby improving the ability to resist flooding stress (Xiao et al., 2018). At present, the mechanism of the effect of DFAA on microbial community diversity is not clear.

Soil metabolites and soil enzyme activities interact with rhizosphere microorganisms and affect plant stress resistance. The concentration of soil metabolites can not only change the physical and chemical properties and microbial activity of soil but can also affect many physiological and biochemical processes at the soil–plant interface, directly or indirectly improving plant stress resistance. Microbial metabolites, soil metabolites and some process products gather in the rhizosphere and interact with each other. The analysis of rhizosphere soil metabolism is helpful to better understand the biological processes of rhizosphere microecosystems (Johns et al., 2017; Rugova et al., 2017). Soil enzymes are an important index to determine soil biological activity. Soil enzyme activity reflects the intensity and direction of various biochemical reaction processes in soil and affects the number of microorganisms, soil metabolism and material cycle of the soil ecosystem. Therefore, the response mechanism of the rhizosphere soil environment to DFAA stress needs to be further studied.

This study was carried out at the Xinxiang Comprehensive Experimental Base of the Chinese Academy of Agricultural Sciences. From the jointing to the tassel flowering stage of maize, the experiment consisted of multiple stages (beginning and end of the drought period, end of the flooding period, 10 days and 20 days after rewatering, harvest period) and multiple combinations (two DFAA groups and one normal irrigation control group). The whole process of DFAA was decomposed, and the dynamic changes in rhizosphere bacteria, metabolites and soil enzyme activity indexes with stress treatment were analyzed. By simulating the two soil moisture conditions of ‘sporadic light rain’ to flood and ‘continuous drought’ to flood, the differences in the rhizosphere bacteria, metabolites and soil enzyme activities of maize in different DFAA groups were identified, and the response mechanism of maize to adapt to DFAA stress was revealed. This study provides new ideas for exploring the potential mechanism of crop yield compensation under DFAA.

## Materials and methods

### Experimental design and sample collection

#### Maize DFAA experiment setting

**Figure 1:**
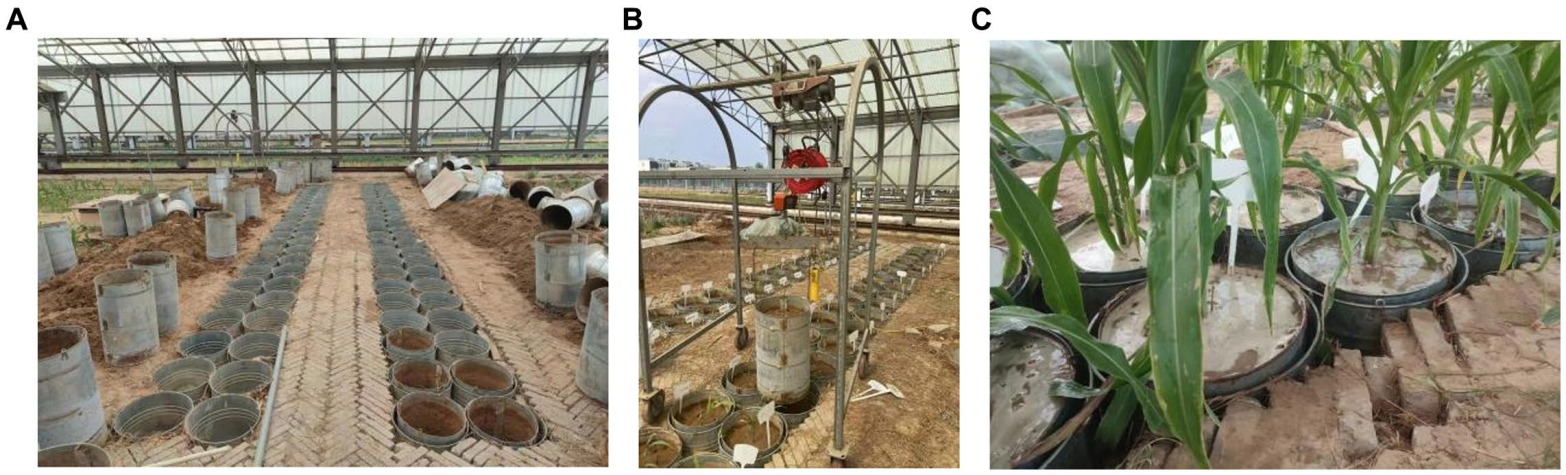
Experimental treatment. **(A–C)** Represent the bucket setting, water control in the dry period and water flooding treatment, respectively.

The experiment was carried out at the Xinxiang Comprehensive Test Base of the Chinese Academy of Agricultural Sciences (35°9’N, 113°47′E). The experimental area is located in Qiliying town, Xinxiang County, Henan Province, which belongs to the warm temperate continental monsoon climate zone, with an average annual temperature of 14°C, precipitation of 548 mm, sunshine duration of 2,399 h, and an average elevation of 78.7 m. The local maize variety Weike 702 (with an approximately 100-day growth period) was selected for the experiment, and the sowing date was June 20. The bucket test method was used. The inner diameter of the bucket was 37 cm, and the height was 60 cm. It was a large-bottomed iron bucket. The test bucket was buried in the soil and covered with a rain shelter to protect it from rain (Figure 1A). During the dry period, the weighing method was used to control the soil moisture in the bucket (Figure 1B). During the flooding period, a 10 cm water layer was maintained in the bucket (Figure 1C). The planting density was 1 plant/barrel. Before planting, compound fertilizer 8 g/barrel (N, P, K contents were 15%) was applied, and no additional fertilizer was applied during the experiment. The soil had a loam texture. The average bulk density of the 0–20 cm soil layer was 1.49 g/cm^3^, and the mass percentage of the field water-holding capacity was 21%.

Factors and levels of drought and flood: Two DFAA groups and one normal irrigation group were set up in the experiment (Table 1), with 6 replicates in each group (6 measuring barrels). Two soil moisture conditions were set up in the drought stage of the DFAA group. DFAA1 was used to simulate ‘sporadic light rain’ to flood, and DFAA2 was used to simulate ‘continuous drought’ to flood. During the flooding period, the barrel water level was observed daily in the morning and evening, and the watering was less than 10 cm. Flooding period continued for 3 days. The experiment started with drought treatment from the jointing stage (July 17), followed by flooding treatment on August 4 and normal irrigation at the end of DFAA treatment (Xiao and Song, 2017). At the beginning and end of the drought period, the end of the flood period, and 10 days and 20 days after rewatering, the rhizosphere soil samples at 0–20 cm depth in the two DFAA groups were collected, and the grain yield of all treatment groups was measured at the harvest period. Summer maize growth period (Table 2).

**Table 1:** Factors and levels of drought and flood.

Treatment	Drought treatment	Drought period	Flooding depth	Flooding period
DFAA1	70% field capacity (maintained)	18 days	10 cm	3 days
DFAA2	70% field capacity (initial) + natural drying	18 days	10 cm	3 days
CK	Normal irrigation			

DFAA – drought-flood abrupt alternation group; CK – control group. 70% field capacity – the soil water content was 70% of the field capacity. Soil moisture was measured by weighing every day during the dry period, and the depth of the water layer was observed every day during the flooding period, which could not reach the predetermined irrigation value. The irrigation mode of the CK group was based on the irrigation system of maize planted in the field: irrigation was performed four times, at the jointing stage, heading stage, flowering stage and milk ripening stage, with an irrigation quota of approximately 40 m^3^/acre.

**Table 2:** Growth period of summer maize.

Treatment	Sowing stage	Seeding stage	Jointing stage	Tasseling stage	Mature stage
DFAA1	June 20th	June 25th	July 17th	August 10th	September 25th
DFAA2	June 20th	June 25th	July 17th	August 10th	September 25th
CK	June 20th	June 25th	July 17th	August 9th	September 17th

The dates in the table are the start of each growth period.

### Maize rhizosphere sample collection

At the beginning and end of the drought period, the end of the flooding period, and 10 days and 20 days after rewatering, the test bucket was opened on one side and used to obtain the rhizosphere samples. The fine roots of maize at 0–20 cm depth were cut off, and the soil samples attached to the fine roots were collected. The sample was the rhizosphere soil of maize (generally considered less than 5 mm from the root system). The flooding treatment group was sampled, and the water layer on the surface of the measuring barrel was extracted by a suction air pressure water gun to ensure that there was no visible water when the soil was collected. Disposable gloves were worn throughout the sampling process.

### DNA extraction, PCR amplification and Illumina sequencing of maize rhizosphere soil

Total microbial genomic DNA was extracted from soil samples using the E.Z.N.A.^®^ soil DNA Kit (Omega Biotek, Norcross, GA, USA) according to the manufacturer’s instructions. The quality and concentration of DNA were determined by 1.0% agarose gel electrophoresis and a NanoDrop^®^ ND-2000 spectrophotometer (Thermo Scientific Inc., USA), and the DNA was kept at −80°C prior to further use. The hypervariable region V3-V4 of the bacterial 16S rRNA gene was amplified with primer pairs 338F (5’-ACTCCTACGGGAGGCAGCAG-3′) and 806R (5’-GGACTACHVGGGTWTCTAAT-3′) by an ABI GeneAmp^®^ 9700 PCR thermocycler (ABI, CA, USA). The PCR mixture included 4 μL of 5 × Fast Pfu buffer, 2 μL of 2.5 mM dNTPs, 0.8 μL of each primer (5 μM), 0.4 μL of Fast Pfu polymerase, 10 ng of template DNA, and ddH_2_O to a final volume of 20 μL. The PCR amplification cycling conditions were as follows: initial denaturation at 95°C for 3 min, followed by 27 cycles of denaturing at 95°C for 30 s, annealing at 55°C for 30 s, extension at 72°C for 45 s, and a single extension at 72°C for 10 min. All samples were amplified in triplicate. The PCR product was extracted from a 2% agarose gel and purified using the AxyPrep DNA Gel Extraction Kit (Axygen Biosciences, Union City, CA, USA) according to the manufacturer’s instructions and quantified using a Quantus^™^ Fluorometer (Promega, USA). Purified amplicons were pooled in equimolar amounts and paired-end sequenced on an Illumina MiSeq PE300 platform/NovaSeq PE250 platform (Illumina, San Diego, USA) according to the standard protocols by Majorbio Bio-Pharm Technology Co. Ltd. (Shanghai, China).

### Detection and identification of soil metabolites

Soil samples (50 mg) were accurately weighed, and the metabolites were extracted using a 400 μL methanol:water (4:1, v/v) solution with 0.02 mg/mL L^−2^-chlorophenylalanin as an internal standard. The mixture was allowed to settle at −10°C and treated with a Wonbio-96c high-throughput tissue crusher (Shanghai Wanbo Biotechnology Co., Ltd.) at 50 Hz for 6 min, followed by ultrasound at 40 kHz for 30 min at 5°C. The samples were placed at −20°C for 30 min to precipitate proteins. After centrifugation at 13000 *g* at 4°C for 15 min, the supernatant was carefully transferred to sample vials for LC–MS/MS analysis. As a part of the system conditioning and quality control process, a pooled quality control sample (QC) was prepared by mixing equal volumes of all samples. The QC samples were produced and tested in the same manner as the analytical samples, which helped to represent the whole sample set, and were injected at regular intervals (every 5–15 samples) to monitor the stability of the analysis. Chromatographic separation of the metabolites was performed on an ExionLCTMAD system (AB Sciex, USA). After UPLC-TOF/MS analyses, the raw data were imported into Progenesis QI 2.3 (Nonlinear Dynamics, Waters, USA) for peak detection and alignment. The preprocessing results generated a data matrix that consisted of the retention time (RT), mass-to-charge ratio (m/z) values, and peak intensity. Metabolic features detected in at least 80% of any set of samples were retained. After filtering, minimum metabolite values were imputed for specific samples in which the metabolite levels fell below the lower limit of quantitation, and each metabolic feature was normalized by summing. The internal standard was used for data QC (reproducibility), and metabolic features with a relative standard deviation (RSD) of QC > 30% were discarded. Following normalization procedures and imputation, statistical analysis was performed on log-transformed data to identify significant differences in metabolite levels between comparable groups. The mass spectra of these metabolic features were identified by using accurate mass, MS/MS fragment spectra and isotope ratio differences by searching reliable biochemical databases, such as KEGG,^1^ HMDB,^2^ and the Metlin database.^3^

### Soil enzyme activity detection method

Soil catalase (S-CAT), dehydrogenase (S-DHA), urease (S-UE), sucrase (S-SC) and acid phosphatase (S-ACP) activities were detected. S-CAT is an important enzyme in soil microbial metabolism and plays an important role in the H_2_O_2_ clearance system. H_2_O_2_ has a characteristic absorption peak at 240 nm. By measuring the change in the absorbance of the solution at this wavelength after reacting with the soil, the activity of soil catalase can be determined. The activity of S-DHA can reflect the active microbial biomass in the soil system and its degradation activity of organic matter and can be used as a degradation performance index of soil microorganisms. The hydrogen receptor 2,3,5-triphenyl tetrazolium chloride (TTC) is reduced to triphenyl formazone (TF) after accepting hydrogen during cell respiration. The maximum absorption peak was at 485 nm. The absorbance value was determined by spectrophotometry at 485 nm, and the soil dehydrogenase activity was obtained. S-UE is a hydrolase that can catalyze the decomposition of urea to produce ammonia and carbonic acid with high specificity. The NH_3_-N produced by urease hydrolysis of urea can react with sodium hypochlorite and phenol in a strong alkaline medium to produce the water-soluble blue dye indophenol blue. The product has a characteristic absorption peak at 630 nm, and the activity of soil urease can be characterized by the change in absorbance. S-SC can hydrolyse sucrose into corresponding monosaccharides and be absorbed by the body. Its enzymatic products are closely related to the contents of organic matter, nitrogen and phosphorus in soil, the number of microorganisms and the soil respiration intensity, which can be used as an important index to evaluate soil fertility. Soil sucrase catalyzes the degradation of sucrose to reducing sugars, which further react with 3,5-dinitrosalicylic acid to form brown–red amino compounds. The product has a characteristic absorption peak at 540 nm, and the activity of soil sucrase can be characterized by the change in the absorption value. S-ACP is an enzyme that catalyzes the mineralization of soil organic phosphorus compounds. Its activity directly affects the decomposition and transformation of organic phosphorus in soil and its bioavailability. It is an important index to evaluate the direction and intensity of soil phosphorus biotransformation. Soil phosphatase is significantly affected by soil carbon, the nitrogen content, the available phosphorus content and pH. It is usually divided into alkaline, neutral and acid phosphatase according to its optimum pH range. Soil acid phosphatase can catalyze the hydrolysis of disodium phenyl phosphate to produce phenol and disodium hydrogen phosphate in an acidic environment. The activity of soil acid phosphatase can be characterized by measuring the amount of phenol produced.

### Data processing and statistical analysis

#### Analysis of microbial community diversity sequencing data

The ACE index, Chao1 index and Shannon index of alpha diversity were calculated by mothur software,^4^ and the difference in alpha diversity between groups was analyzed by Student’s t test. The species composition of different treatment groups at each phylum level was calculated, and a community histogram was drawn to analyze the dominant species and their relative abundance at the phylum level.

#### Association analysis of differential bacteria and soil metabolites between the two DFAA groups

The Welch T test was used to test the hypothesis related to species between microbial communities at each genus level in different treatment groups to evaluate the significant level of the species abundance difference and to obtain significantly different species between groups. Correlation analysis was performed according to the degree of variation in metabolite composition between different treatment groups and the relative abundance index of dominant species at the microbial genus level.

#### Calculation of soil enzyme activity

The S-CAT activity was measured as the degradation of 1 mmol H_2_O_2_ into an enzyme activity unit per gram of dry soil sample per day. The S-DHA activity was measured as the production of 1 μg TF per gram of soil sample per day at 37°C as an enzyme activity unit. The S-UE activity was measured as the production of 1 μg NH_3_-N per gram of soil sample per day as an enzyme activity unit. The S-SC activity was measured as the production of 1 mg reducing sugar per gram of soil sample per day as an enzyme activity unit. The S-ACP was measured as the production of 1 nmol phenol per gram of soil per day at 37°C as an enzyme activity unit. The general formula for enzyme activity calculation is as follows:


$$U=\frac{D\times 10^{3}\times \left(\Delta A_{1}-\Delta A_{2}\right)\times V_{t}}{e\times V_{s}\times d}$$

where D represents the dilution multiple; ΔA_1_ represents the change in the absorbance value of the sample; ΔA_2_ represents the change in the absorbance value of the blank control; V_t_ represents the total volume of the reaction, mL; e represents the molar absorbance value, 6.22 × 10^−3^ mol/L·cm^−2^; V_s_ represents the enzyme liquid volume, mL; and d represents the light path of the cuvette, 1 cm.

## Results and analysis

### Effects of DFAA stress on maize rhizosphere bacteria

**Figure 2:**
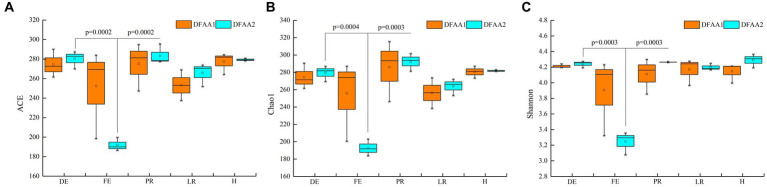
Alpha diversity index. **(A–C)** ACE index, Chao1 index and Shannon index, respectively. DE, FE, PR, LR, and H represent the dry period, flooding period, early rewatering period, late rewatering period and end of the harvesting period, respectively.

From Figure 2, it can be seen that the alpha diversity index of DFAA1 and DFAA2 rhizosphere soil bacteria did not show significant differences in the five stages of DFAA. The diversity of maize rhizosphere bacteria in the flooding stage of DFAA2 was significantly lower than that in other stages, indicating that the ‘continuous drought’ to flooding in the previous 18 days reduced the diversity of rhizosphere bacteria. The reason may be that under DFAA conditions, the rapid closure of soil air channels led to a decrease in aerobic flora.

**Figure 3:**
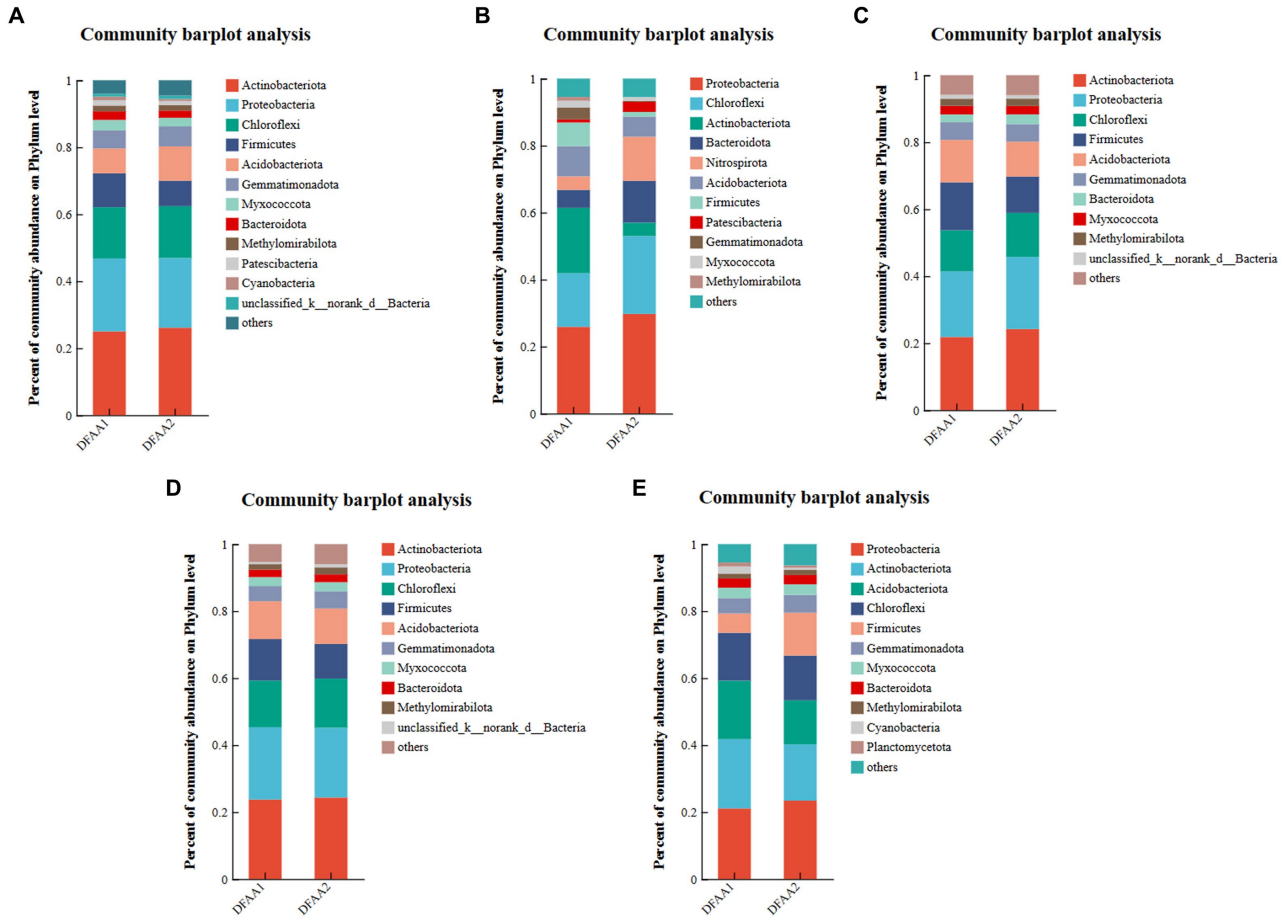
Microbial community composition. **(A–E)** Represent the rhizosphere microbial community composition of maize during the drought period, flooding period, early rewatering period, late rewatering period and harvesting period, respectively. Species classification database: silva138/16s_bacteria; the combined abundance of ‘others’ accounted for less than 1%, the same below.

According to Figure 3, Actinobacteria was the most dominant phylum in the two DFAA groups in the drought period, early rewatering period and late rewatering period, and Proteobacteria was the most dominant phylum in the flood period and harvest period. The relative abundances of Proteobacteria (29.73%) and Chloroflexi (23.26%) in the DFAA2 group during the flooding period were higher than those of other phyla. The dominant phyla in the DFAA1 and DFAA2 groups during the drought period, prerewatering period, postrewatering period, and harvest period and in the DFAA1 group during the flooding period were Actinobacteria and Proteobacteria (the sum of the two accounted for nearly 50%). Actinobacteriota was widely distributed in the DFAA groups in the drought period, rewatering period and harvest period. Members of Actinobacteriota are aerobic and can produce a variety of beneficial metabolites (Yang et al., 2022). They can also construct a symbiotic system with roots, improve the rhizosphere soil environment and promote plant growth and development (Ning et al., 2019). Proteobacteria was the dominant phylum in the whole stage of the two DFAA groups. Because this phylum consists of facultative or obligate anaerobic bacteria, it contains chemoautotrophic and chemoheterotrophic microorganisms, including a variety of metabolic species. Changes in its composition and quantity have complex effects on the growth of maize roots (Li et al., 2020; Wu et al., 2021). The proportion of Chloroflexi in the DFAA2 group increased during the flooding period. Members of this phylum perform both aerobic respiration and anaerobic respiration, as well as extremely diverse nutritional methods such as photoautotrophy, photoheterotrophy, chemoautotrophy, chemoheterotrophy and mixed nutrition, and some phototrophic groups can form green bodies that store photosynthetic pigments so that they can store energy through photosynthesis (Ward et al., 2018). Therefore, it may enhance the ability of the DFAA2 group to adapt to flooding.

**Figure 4:**
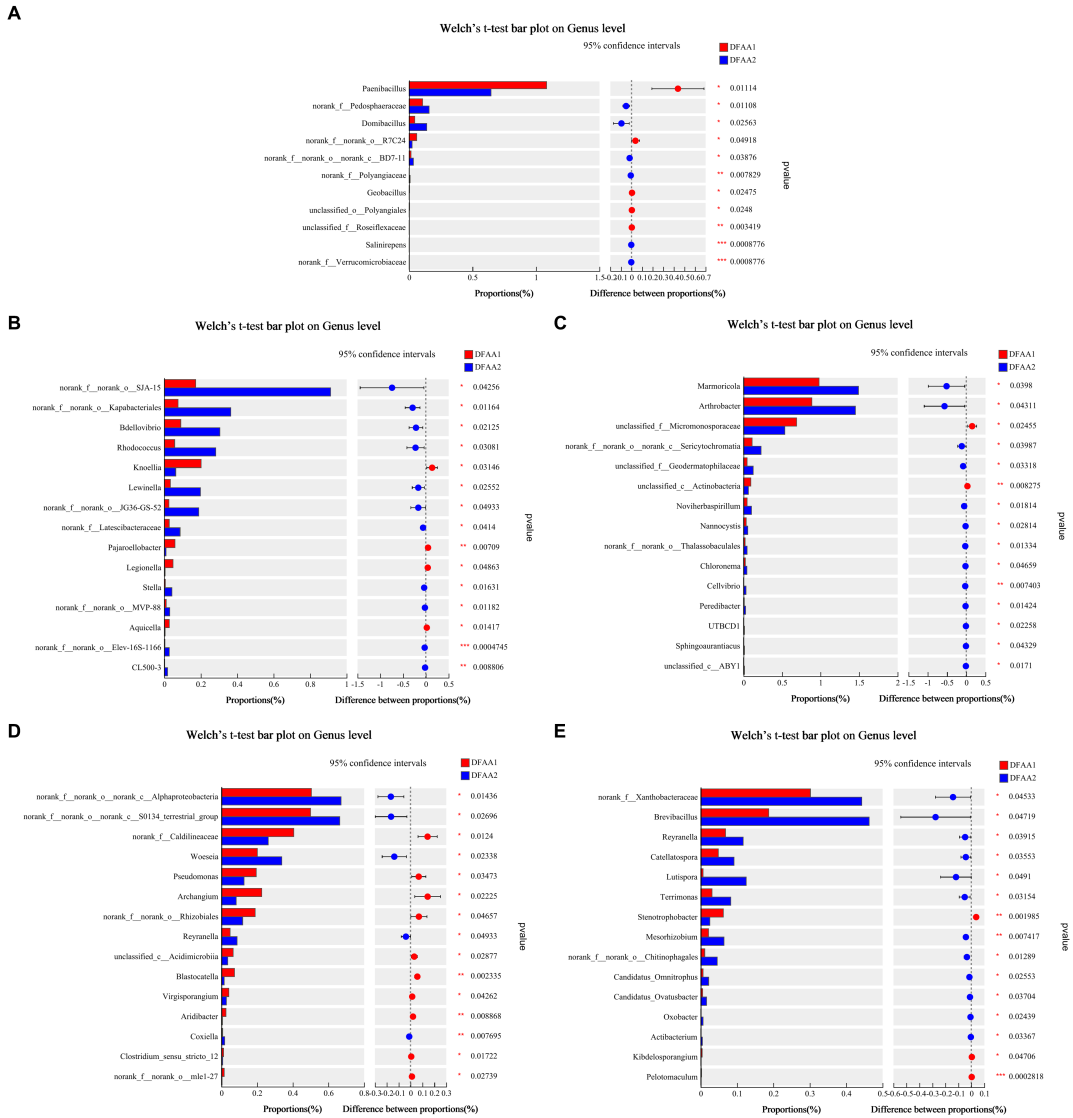
Significance testing of differences between groups. **(A–E)** Represent the average relative abundance of maize rhizosphere microbial communities at the genus level during the drought period, flooding period, early rewatering period, late rewatering period and harvesting period, respectively.

The differences in rhizosphere microbial species in the two DFAA groups at the genus level were further analyzed. From Figure 4, the relative abundance of *Paenibacillus* in the DFAA1 group was significantly higher than that in the DFAA2 group in the drought period, while *SJA-15* in the flooding period, *Arthrobacter* in the early stage of rewatering, *Alphaproteobacteria* in the late stage of rewatering, and *Brevibacillus* in the harvest period were enriched in the DFAA2 group. Because the genus *Paenibacillus* stimulates plant growth (Grady et al., 2016), the soil environment under ‘sporadic light rain’ for the first 18 days was more conducive to the growth of maize than the soil environment under ‘continuous drought’. *SJA-15* belongs to Chloroflexi, which has the characteristics and metabolic characteristics of this phylum. Compared with ‘sporadic light rain’ to flooding, *SJA-15* was more likely to be enriched in the rhizosphere soil from the ‘continuous drought’ to flooding, which as beneficial for improving the flooding tolerance of maize. *Arthrobacter*, *Alphaproteobacteria* and *Brevibacillus* were soil microbial genera that experienced DFAA stress, and the results showed that the enrichment was more significant in the soil from the ‘continuous drought’ to flooding. It has been reported that *Arthrobacter* has the characteristics of promoting plant growth activity (Fernández-González et al., 2017). *Alphaproteobacteria* will survive in a low-nutrient environment in a symbiotic way with plants (such as rhizobia) (Chen et al., 2022). *Brevibacillus* can induce plant disease resistance and promote the absorption of nutrients by plants (Yang and Yousef, 2018). Therefore, the above bacteria played a beneficial role in promoting soil remediation after the ‘continuous drought’ to flooding.

### Association analysis between soil metabolites and differential bacteria in the two DFAA groups

**Figure 5:**
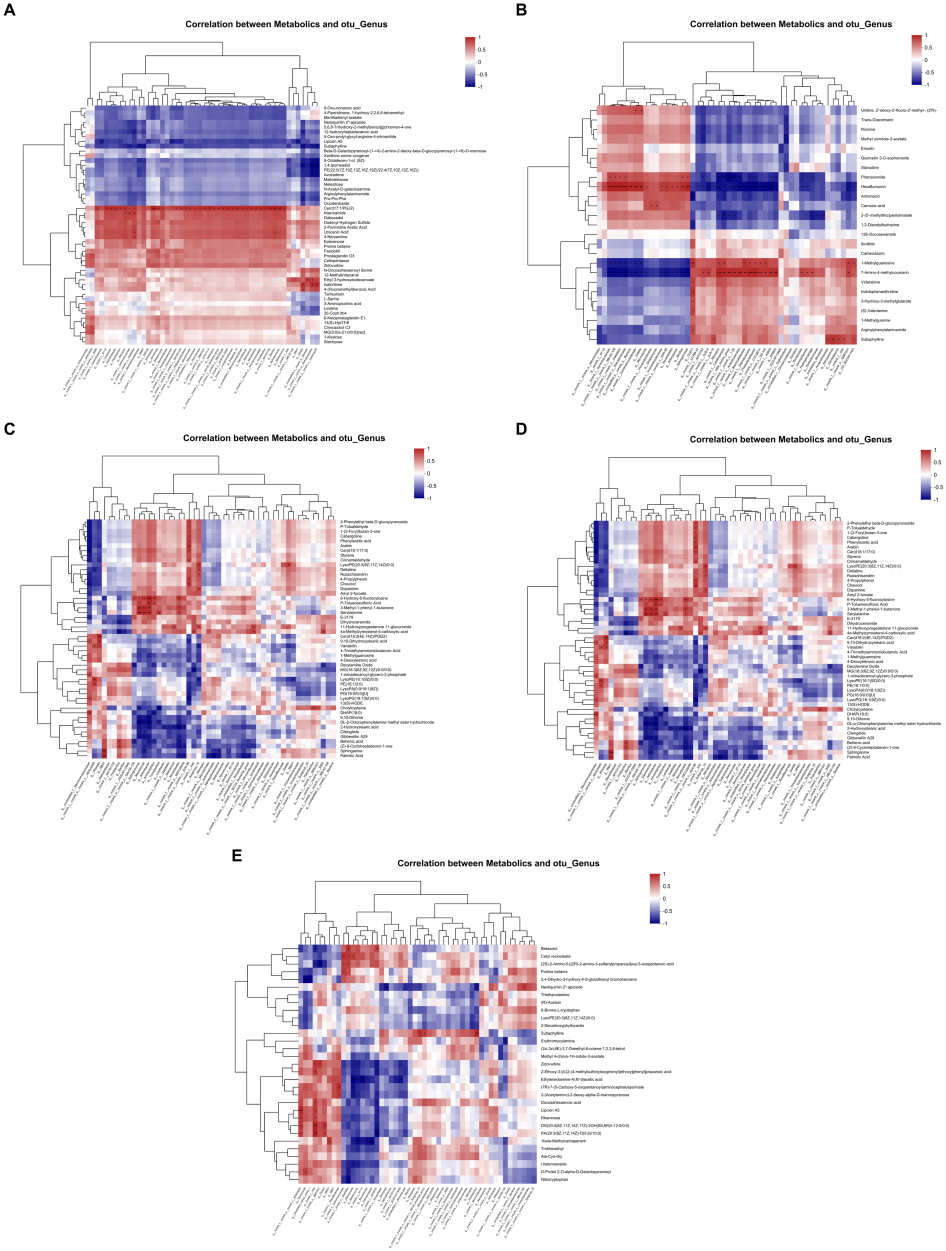
Correlation heatmap of rhizosphere bacteria and the metabolome. **(A–E)** Represent the drought period, flooding period, early rewatering period, late rewatering period and harvesting period, respectively. The right side of the figure is the name of the metabolite, the bottom is the name of the rhizosphere microorganism, each grid in the figure represents the correlation between the two attributes, and the different colors represent the magnitude of the correlation coefficient between the attributes.

A hierarchical clustering algorithm was used to calculate the correlation between the top 50 soil metabolic groups and rhizosphere bacteria, as shown in Figure 5. It was found that there was a significant positive correlation between the soil metabolite Cer(d17:1/PGJ2) and *Paenibacillus,* the differential genus of the two DFAA groups during the drought period. During the flooding period, the soil metabolite subaphylline was significantly positively correlated with the differential genus *SJA-15*. At the early stage of rewatering, the soil metabolites 6-hydroxy-5-fluorocytosine, P-toluenesulfonic acid, 3-methyl-1-phenyl-1-butanone and serine were significantly positively correlated with the differential genus *Arthrobacter*. In the late stage of rewatering, the soil metabolite 28-homobrassinolide brivaracetam MG (10:0/0:0/0:0) was significantly positively correlated with the differential genus *Alphaproteobacteria*. No soil metabolites significantly associated with the differential genus *Brevibacillus* were detected during the harvest period. The above metabolite, Cer(d17:1/PGJ2), is a bioactive lipid that promotes the proliferation or apoptosis of maize root cells during the drought period (Grady et al., 2016; Matsufuji et al., 2018). Subaphylline is a biogenic amine that enhances the tolerance of plants to biotic or abiotic stresses under flooding conditions (Killiny and Nehela, 2020; Su et al., 2022). 6-Hydroxy-5-fluorocytosine, P-toluenesulfonic acid, etc., are hydrocarbon compounds that have antifungal, anti-insect, catalytic and other effects in the early stage of rehydration. In addition, 28-homobrassinolide brivaracetam MG (10:0/0:0/0:0) is a steroid hormone that has a protective effect on plants in the late stage of rewatering and can reduce the effects of natural conditions (such as cold, drought, high temperature or salinity) on plant stress (Kaur et al., 2018).

### Effects of DFAA stress on soil enzyme activities in the maize rhizosphere

**Figure 6:**
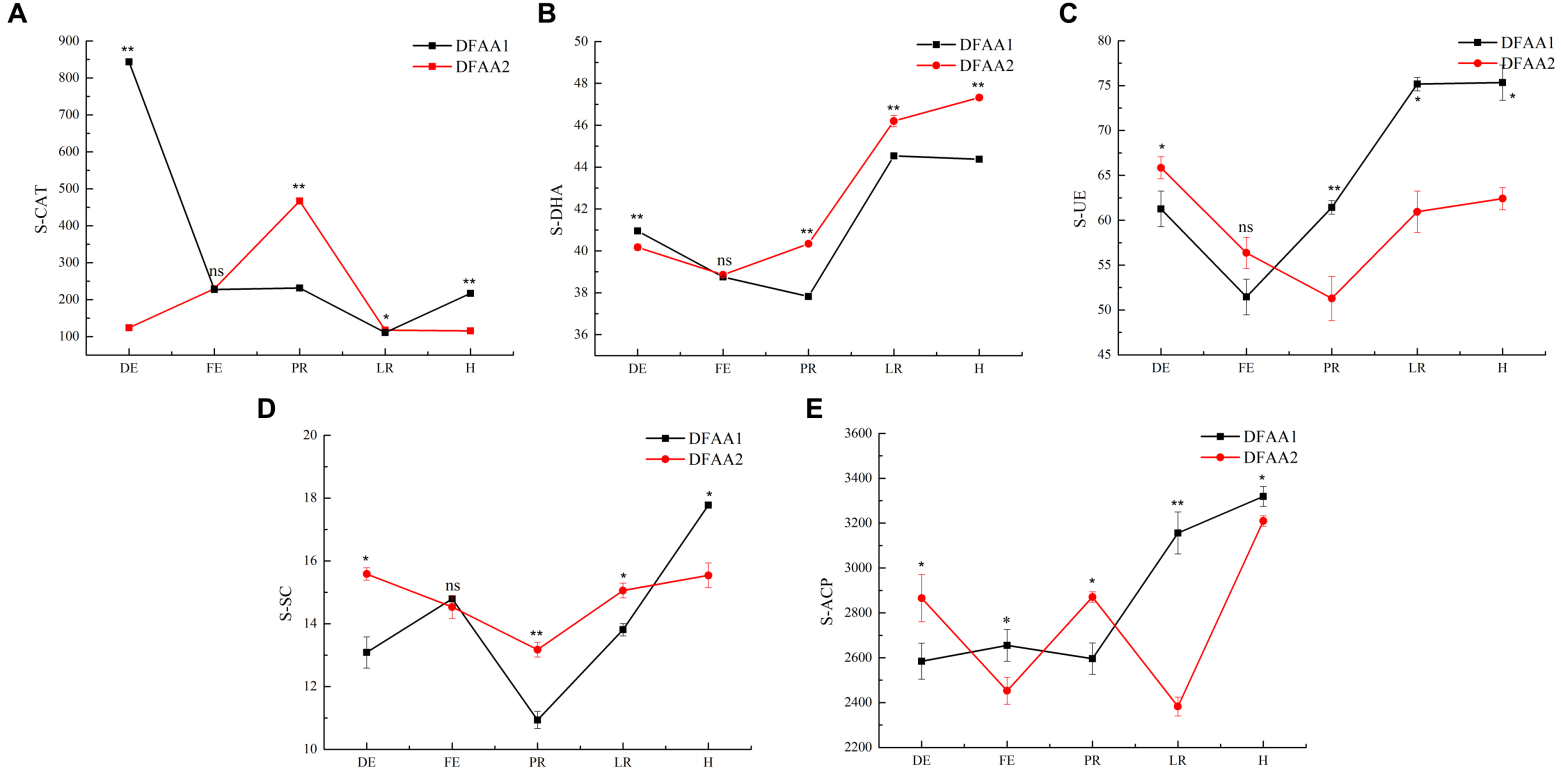
Enzyme activity in the rhizosphere. **(A–E)** The activities of S-CAT, S-DHA, S-UE, S-SC, and S-ACP are shown. The unit of soil enzyme activity is U/g soil sample.

Figure 6 shows that the activities of soil catalase (S-CAT), dehydrogenase (S-DHA), urease (S-UE), sucrase (S-SC) and acid phosphatase (S-ACP) in the two DFAA groups during the five stages showed significant differences. In the drought period, the S-CAT and S-DHA in the DFAA1 group were higher than those in the DFAA2 group, indicating that the ‘sporadic light rain’ in the early 18 days greatly reduced the damage caused by hydrogen peroxide to the plants, and it was easier to catalyze and decompose organic substances and promote the growth and reproduction of soil bacteria (Cui et al., 2020). During the flooding period, the S-ACP of the DFAA1 group was higher than that of the DFAA2 group, and there was no significant difference in the other four enzymes, indicating that the root and rhizosphere bacteria of maize under the ‘sporadic light rain’ to flooding conditions secreted more acid phosphatase to decompose organic phosphorus in the soil and increase the nutrient and energy consumption in the plant, thereby reducing the plant’s flooding tolerance (Chen and Liang, 2020). In the early stage of rewatering, except for S-UE, the activities of S-CAT, S-DHA, S-SC and S-ACP in the soil of the DFAA2 group were higher than those of the DFAA1 group, indicating that ‘continuous drought’ to flooding promoted the decomposition and oxidation of carbon and organic phosphorus substances in the soil; carbon metabolism released a large amount of energy and promoted the recovery and reproduction of bacteria in the soil (Shu et al., 2018). At the late stage of rewatering, the activities of S-CAT, S-DHA and S-SC in the soil of the DFAA2 group were significantly higher than those in the DFAA1 group, indicating that the oxidoreductases in the soil under the ‘continuous drought’ to flooding conditions continued to eliminate the effect of hydrogen peroxide on plants, and sucrase promoted sucrose degradation, organic matter mineralization and plant nutrient absorption (Zhang et al., 2022). At the harvest stage, the activities of S-CAT, S-UE, S-SC and S-ACP in the DFAA1 group were higher than those in the DFAA2 group except for S-DHA, indicating that the conversion of ‘sporadic light rain’ to flooding had a stronger enzymatic effect on the decomposition and oxidation of organic matter, nitrogen and phosphorus in the soil and a stronger ability to remove hydrogen peroxide (Feng et al., 2020). In summary, the decomposition of organophosphorus compounds, consumption of nutrients and energy by the plant, and the reduction in the plant flooding tolerance in the DFAA1 group at the flooding stage of DFAA stress were not conducive to plant survival and later growth recovery, while the DFAA2 group continued to decompose and oxidize nitrogen, phosphorus and potassium during the rewatering period after DFAA stress, providing energy for the reproduction of soil bacteria and plant growth.

## Discussion

### Response of the rhizosphere soil environment to DFAA stress

Under DFAA conditions, crops are affected by the dual stress factors of drought and flooding. There is an effect of the interaction between different drought and flood stress combinations on rhizosphere bacteria. Under multiple environmental stresses, crops and rhizosphere bacteria release more metabolites and unknown compounds to improve the stress resistance and survival ability of crops. At present, more attention is given to single drought and flood stress (Coutinho et al., 2016; Xiao et al., 2018; Xu et al., 2018; Varoquaux et al., 2019; Xu and Coleman-Derr, 2019; Xu et al., 2021). The mechanism of the effect of drought and flood stress on rhizosphere bacteria, soil metabolites and soil enzyme activity is not clear. This study showed that the response of the rhizosphere soil environment to DFAA stress was different in the two DFAA groups. Under the condition of ‘sporadic light rain’ in the previous 18 days, the abundance of *Paenibacillus* was higher, and the bioactive lipid metabolites with a significant positive correlation had the potential to promote the growth of maize and the proliferation or apoptosis of maize root cells. In addition, ‘sporadic light rain’ greatly alleviated the damage caused by hydrogen peroxide to maize, catalyzed and decomposed organic matter, and promoted the growth and reproduction of soil bacteria. Therefore, the DFAA1 group was more suitable for plant growth before flooding. Under the condition of ‘continuous drought’ to flooding, Chloroflexi, *SJA-15* bacteria and the biogenic amine metabolite subaphylline during the flooding period may have potential functions to help plants adapt to the environment of DFAA stress (Nierychlo et al., 2019; Xian et al., 2020). From rehydration to harvest, *Arthrobacter*, *Alphaproteobacteria* and *Brevibacillus*, as well as hydrocarbons and steroid hormone metabolites, may have the potential to promote plant growth activity, hypoxia tolerance, and disease resistance and reduce biotic and abiotic stresses. In addition, the activities of S-CAT, S-DHA and S-SC in the soil during the rehydration period were higher, which promoted sucrose degradation, organic matter mineralization and nutrient absorption by maize, reduced the damage caused by hydrogen peroxide to maize and were conducive to the recovery and reproduction of bacteria in the soil. Therefore, the DFAA2 group was more focused on repairing the soil environment after flooding.

### Potential effects of soil environmental changes on maize yield under DFAA stress

**Figure 7:**
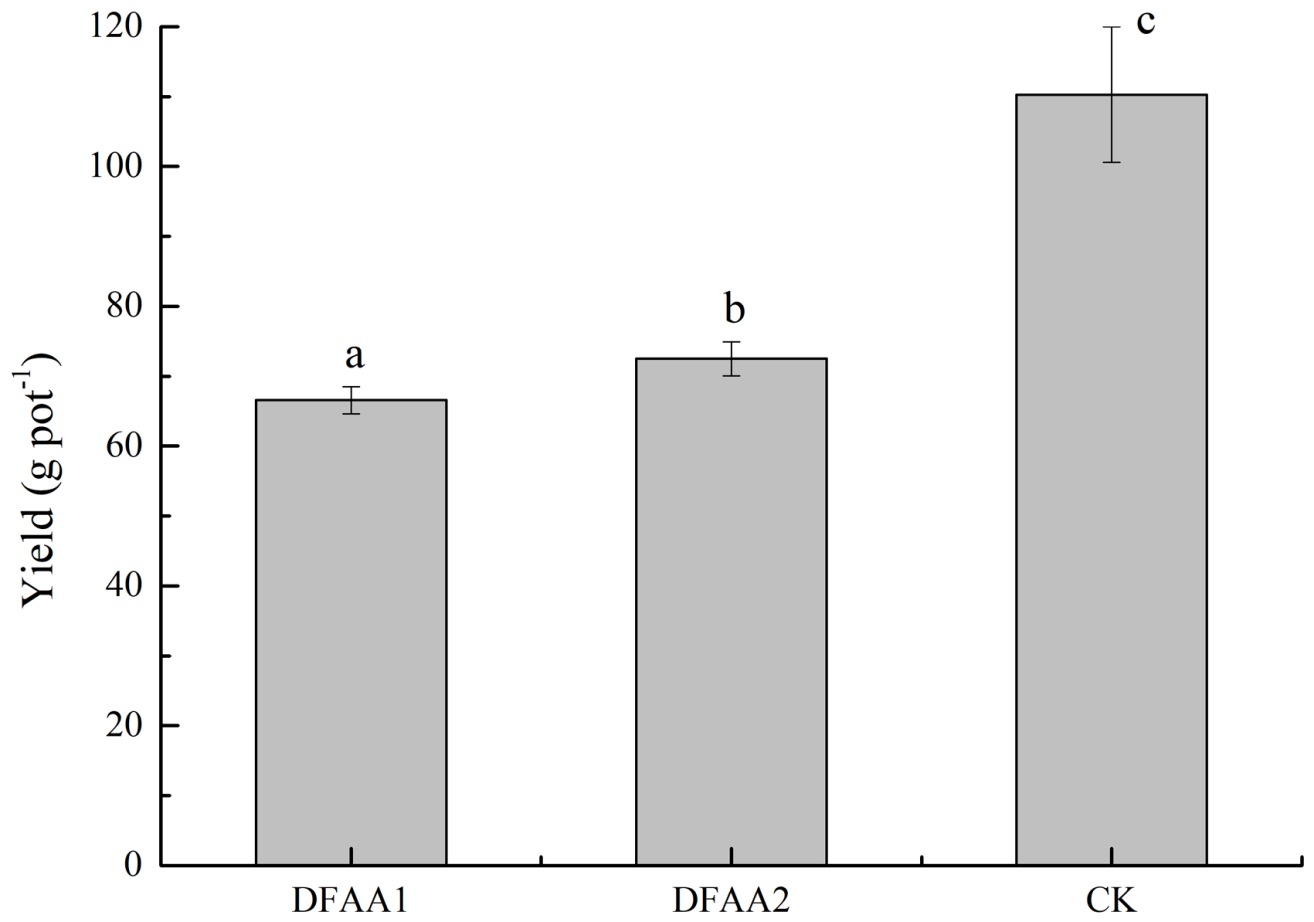
Yield of the drought-flood abrupt alternation group and the control group.

Abnormal global climate change has led to DFAA occurs frequently, which seriously threaten China’s water security and food security. It is an urgent problem to be solved in agricultural production to reveal the response of the soil environment and its influence on the stress resistance characteristics of crops under extreme climate change and to formulate reasonable countermeasures for farmland disaster reduction and reduce yield loss. At present, more attention has been given to the response of the crop yield, root system and physiological growth index to DFAA. Studies have pointed out that there are compensation phenomena in the crop yield, root systems, stems, leaves and photosynthesis under DFAA stress (Gao et al., 2019; Huang et al., 2019; Bi et al., 2020; Zhu et al., 2020), but an explanation of the stress response mechanism of crops under DFAA stress from the perspective of the soil environment has not been carried out. The results of this study showed that there was a significant difference in maize yield under DFAA condition and normal irrigation condition. The average yield reduction rate was 36.95% in the DFAA group, 39.64% in the DFAA1 group and 34.27% in the DFAA2 group (Figure 7). The reason for yield reduction of the two DFAA groups was due to the combined effects of drought, flooding and soil microorganisms. Therefore, in the next study, the accurate measurement of oxygen in the bucket under flooding condition can be increased. The ‘sporadic light rain’ to flooding caused greater damage to maize yield, while the ‘continuous drought’ to flooding caused yield compensation, which was related to the improvement of flooding tolerance of maize by Chloroflexi, *SJA-15* and biogenic amine metabolites. These rhizosphere bacteria and soil metabolites may have the potential function of helping plants adapt to the DFAA environment. In addition, this study indicates that the period of DFAA occurrence will also have an impact. In this study, the DFAA experiment was set from the jointing stage to the heading stage of maize. The flooding occurred on August 4, and the beginning and end of rewatering (August 7 to August 27) were the critical periods of flowering and grain filling. The recovery of the soil environment, such as microbial function and soil enzyme activity, at this stage plays a key role in grain filling, grain weight and yield formation (Gao et al., 2022).

## Conclusion

In this study, two scenarios of ‘sporadic light rain’ to flooding and ‘continuous drought’ to flooding, were set up to analyze the changes in rhizosphere bacteria, soil metabolites and soil enzyme activities in different DFAA groups and to reveal the response of the maize rhizosphere soil environment to DFAA stress to provide new ideas for exploring the potential mechanism of crop yield compensation under DFAA. The study suggests that there is a yield compensation phenomenon in the conversion of ‘continuous drought’ to flooding compared with ‘sporadic light rain’. The reason may be related to the improvement in the flooding tolerance of maize by the dominant bacteria Chloroflexi, differential bacterium *SJA-15* between the two groups and biogenic amine metabolites. This study has shown that ‘sporadic light rain’ in the first 18 days greatly reduced the damage caused by hydrogen peroxide to corn in the soil, and it was easier to catalyze and decompose organic substances and promote the growth and reproduction of soil bacteria. However, during the flooding period, the roots and rhizosphere bacteria secreted more S-ACP to release organophosphorus compounds in the soil for plant growth, resulting in the consumption of nutrients and energy in the plant and a reduction in the flooding tolerance of the plant. The limitation of the research is that it does not consider the change process of the maize growth index, dry matter quality index, leaf endogenous hormones, antioxidant enzymes, osmotic adjustment substances and other resistance indexes under drought-flood abrupt alternation. Therefore, the way in which crops improve their resistance to adapt to DFAA stress needs further study.

## Data availability statement

The original contributions presented in the study are included in the article/Supplementary material, further inquiries can be directed to the corresponding authors.

## Author contributions

YG: Conceptualization, Data curation, Funding acquisition, Methodology, Writing – original draft. YZ: Data curation, Writing – original draft. PL: Conceptualization, Funding acquisition, Writing – review & editing. XQ: Conceptualization, Writing – original draft, Writing – review & editing.

## Footnotes

^1^ https://www.kegg.jp/kegg/pathway.html

^2^ http://www.hmdb.ca/

^3^ https://metlin.scripps.edu/

^4^ http://www.mothur.org/wiki/Calculators
