# Supplementary material for: Responses of the maize rhizosphere soil environment to drought-flood abrupt alternation stress
Source: Front Microbiol. 2023 Dec 14;14:1295376. doi: 10.3389/fmicb.2023.1295376 (PMC10760638; doi:10.3389/fmicb.2023.1295376)
Supplement: Supplementary file 1 [file Data_Sheet_1.docx]

Appendix: Calculation method for the microbial alpha diversity index

ACE Index:

$$S_{ACE}=\left\{ \begin{aligned} S_{abund}+\frac{S_{rare}}{C_{ACE}}+\frac{n_{1}}{C_{ACE}}\hat{\gamma}_{ACE}^{2}，for\hat{\gamma}_{ACE}<0.80 \\ S_{abund}+\frac{S_{rare}}{C_{ACE}}+\frac{n_{1}}{C_{ACE}}\tilde{\gamma}_{ACE}^{2}，for\hat{\gamma}_{ACE}\geq0.80 \end{aligned} \right.$$

where

$$N_{rare}=\sum_{i=1}^{abund} in_{i}，C_{ACE}=1-\frac{n_{1}}{N_{rare}}$$

$$\hat{\gamma}_{ACE}^{2}=max\left[ \frac{S_{rare}}{C_{ACE}}\frac{\sum_{i=1}^{abund} i(i-1)n_{i}}{N_{rare}(N_{rare}-1)}-1, 0 \right]$$

$$\tilde{\gamma}_{ACE}^{2}=max\left[ \hat{\gamma}_{ACE}^{2}\left\{ 1+\frac{N_{rare}(1-C_{ACE})\sum_{i=1}^{abund} i(i-1)n_{i}}{N_{rare}(N_{rare}-C_{ACE})} \right\}, 0 \right]$$

$n_{i}$ indicates the number of OTUs in the article i sequence;

$S_{rare}$ indicates the number of OTUs with an "abund" bar sequence or the number of OTUs less than "abund".

$S_{abund}$ indicates the number of OTUs higher than the abundance bar sequence.

abund indicates the threshold of the Advantaged OTUs, which is 10 by default.

Chao1 Index:

$$S_{Chao1}=S_{\mathrm{obs}}+\frac{n_{1}(n_{1}-1)}{2(n_{2}+1)}$$

where

$S_{Chao1}$ represents the estimated OTU number;

$S_{\mathrm{obs}}$ represents the number of OTUs actually observed;

$n_{1}$ indicates the number of OTUs containing only one sequence (such as "singletons");

$n_{2}$ indicates the number of OTUs containing only two sequences (as in "doubletons").

Shannon index:

$$H_{Shannon}=-\sum_{i=1}^{S_{obs}} \frac{n_{i}}{N}ln\frac{n_{i}}{N}$$

where

$S_{\mathrm{obs}}$ represents the number of OTUs actually observed.

$n_{i}$ represents the number of sequences contained in the i-th OTU;

N represents all sequence numbers.
